# Methodology for integrating artificial intelligence in healthcare systems: learning from COVID-19 to prepare for Disease X

**DOI:** 10.1007/s43681-021-00111-x

**Published:** 2021-10-19

**Authors:** Petar Radanliev, David De Roure, Carsten Maple, Uchenna Ani

**Affiliations:** 1 Department of Engineering Sciences, Oxford e-Research Centre, University of Oxford, Oxford, UK; 2 WMG Cyber Security Centre, University of Warwick, Coventry, UK; 3 STEaPP, Faculty of Engineering Science, University College London, London, UK

**Keywords:** Artificial intelligence, Healthcare systems, Internet-of-things (IoT), Edge devices, Covid-19, Disease X

## Abstract

Artificial intelligence and edge devices have been used at an increased rate in managing the COVID-19 pandemic. In this article we review the lessons learned from COVID-19 to postulate possible solutions for a Disease X event. The overall purpose of the study and the research problems investigated is the integration of artificial intelligence function in digital healthcare systems. The basic design of the study includes a systematic state-of-the-art review, followed by an evaluation of different approaches to managing global pandemics. The study design then engages with constructing a new methodology for integrating algorithms in healthcare systems, followed by analysis of the new methodology and a discussion. Action research is applied to review existing state of the art, and a qualitative case study method is used to analyse the knowledge acquired from the COVID-19 pandemic. Major trends found as a result of the study derive from the synthesis of COVID-19 knowledge, presenting new insights in the form of a conceptual methodology—that includes six phases for managing a future Disease X event, resulting with a summary map of various problems, solutions and expected results from integrating functional AI in healthcare systems.

## Introduction

The motivation and the problems investigated in this study are the potential algorithmic solutions to the global lack of preparedness for Covid-19 and other Disease X events. The challenges and problems we wanted to overcome in this investigation include the integration of artificial intelligence and modern technologies (through functional algorithms) in healthcare systems. The objectives of the study are to construct a conceptual methodology for preparing and adapting to future waves of Covid-19 and a future ‘Disease X’ event, including measuring risk and predicting the loss, preparing appropriate defence, and suggesting areas for future improvement of AI in healthcare. The significance and contribution of the study are a new conceptual methodology for integrating artificial intelligence and real-time data (e.g. from IoT health devices). The novelty of the proposed methodology is the use of publicly available, open access data sources and insights from low memory IoT devices (e.g. Shodan).

Historically, nothing has killed more humans than infectious diseases. COVID-19 has shown us how vulnerable and how unprepared we are to unanticipated health risks. There could be millions of undiscovered viruses in the world, and the probability of another global pandemic is only a matter of time. A number of pandemics have appeared in the recent past, such as SARS and HIV, but it has been a century since the last major global pandemic—the Spanish flu, which claimed up to 100 million lives, surpassing the death toll of World War One. It could be argued that global pandemics present the highest risk for catastrophic and existential crises, even worse than nuclear war. While nuclear war might stop after few million deaths, pandemics do not discriminate and do not stop. This creates a very strong rationale for increasing our preparedness to control, manage and stop the next global pandemic—also known as Disease X.

Studying COVID-19 presents a unique and rare opportunity—to study a global pandemic in real time, to change and adapt the data collection strategy when new parameters emerge, and to test new solutions. The world has been faced with outbreaks, epidemics, endemics and pandemics many times in the past. However, there are fundamental differences between endemics—which affect a group of particular people or country; outbreaks—which is an increase of endemic cases that present a risk of becoming an epidemic; epidemics—which affect a community, population, or region; and pandemics—which is an epidemic that spread over countries and continents. COVID-19 is a truly global pandemic of a size and magnitude that has not occurred for over 100 years. The last global pandemic was the Spanish flu in 1918. Global pandemics are rare events and should be studied exclusively. One of the crucial differences in our society from the time of the Spanish flu is the rapid rise in technological growth and developments. The emergence and growth of digital healthcare and connected devices is a new element, which was not present during the Spanish flu, and it is something we have exploited strongly during COVID-19. But digital technologies come with inherent digital or cyber risks and that is something we did not contemplate in great detail in our pursuit for solutions to the deadly pandemic.

### Covid-19 has increased the cyber-attack risk surface

Since the rise of Covid-19, the advantages of digital health e.g., online consultations, sharing health data, presented a rationale for an increased investment in Internet-connected health systems and devices, but cybersecurity investment has not risen at a similar rate. This places security of health systems at the top of cybersecurity priority for national crucial infrastructure in 2021 and beyond. Some of the main cyber risks in 2021 could emerge from the threats of nation states and criminals [1], to logistical challenges and disruption of complex supply chains for vaccine distribution [2], and criminal ransomware. It is expected for these cyber dangers to continue to evolve in the future. One of the most concerning future cyber risks is the attacks evolving from locking health data to tampering health data, which will create even more serious risk to patient’s life. Cyber attackers understand that disturbing or impeding the ability to support or maintain critical care can result to a higher probability of being paid.

One cyber risk that is consistently mentioned in cyber security conferences is the risk of attackers hacking IoT medical implants, or insulin pumps, resulting in the death of patients. While such cyber risk is very concerning, the nature of such attacks means that the cyber risk will be localised and isolated. More concerning cyber risks emerge with the growing digitalisation of health system, increasing the risk surface and the risk of cascading effect, which was accelerated by COVID-19. This article constructs some of the potential solutions for preventing the risk of such cascading effect. Apart from cyber risks, this article devises some of the potential solutions for vaccine supply chains. The vaccine supply chains will play a crucial part in managing the pandemic and the complex operations that involve multiple organisations, which make it particularly susceptible to cyber attacks. This motivates this investigation on how we adapt our healthcare system to cope better with Disease X, i.e., any future global pandemics.

### Systematic state-of-the-art literature review

The state-of-the-art literature review builds upon the ‘eleven COVID-19 vaccine challenges’ [3] and the recent systematic literature reviews on COVID-19 pandemic-related supply chain studies [4], but with a greater focus on emerging technologies. Recent studies started investigating a more integrated intelligence as a concept for more objective (and subjective) patient scoring for critical care [5]. Similarly, a detection-based prioritisation is proposed as a concept for recognising patient’s health condition prior to discharge for managing patient care and optimising clinical care [6]. In times of great healthcare urgency, appropriate allocation of resources requires differentiating the health conditions of infected patients and assigning appropriate care with fast and effective treatment [7]. This brings into focus the need for a fast and non-biased detection of future pandemics. One proposed solution is to use machine learning for biological data mining in detecting and diagnosing novel viruses [8]. This approach could prevent future delays in detecting and diagnosing a Disease X event. Such approach could use big data from social media to analyse and classify (i.e. group) the human ‘sentiment’ [9]. Another approach would be to use artificial intelligence to detect and classify medical images and detect abnormalities based on evaluation and benchmarking [10]. What connects all these studies is the need to integrate advanced technologies and algorithms in the prevention and management of future pandemics. In this article, we synthesise knowledge accumulated during COVID, to construct a conceptual methodology for integrating artificial intelligence in healthcare systems—that can be used to prepare for a future Disease X event.

### Research questions

This article addresses existential questions emerging from the COVID-19 pandemic. The questions driving this article are: How do we prepare for future global pandemics, i.e. Disease X with minimal consequences? How do we adapt our healthcare system to cope better to future global pandemics? How do we reduce the various risks from a future Disease X?

## Hide or evolve: two strategies for coping with global pandemics

The COVID-19 pandemic has triggered existential questions and tests healthcare systems to their limits globally. The global response in the first wave can be characterised as a) the primal methods of isolation and distancing learnt from past pandemics; and b) the digital and technology-driven transformations, based on big data analytics and artificial intelligence (AI) for smart healthcare. The second approach leads towards an integrated concept of healthcare systems, enabling flexibility to adapt quickly to changes, promoting faster adoption of new solutions. AI has been used in hospital administration and operations in the past, but mostly in low-risk operations such as reading medical images. Since the rise of COVID-19, AI has also been used in critical healthcare systems to track the pandemic and estimate the risk of death [11].

This trend is expected to continue and evolve into a system of wearables and biomedical devices integrated with AI, transforming clinical research and virtual healthcare. This AI-enhanced transformation will challenge the traditional healthcare systems with problems they have never faced before, increasing the cyber-attack surface and escalating the cyber-risk levels. For example, there is a risk of biased data and high latency in training AI [12]. Another such challenge is integrating cybersecurity and machine learning experts and big data scientists in the forefront of digital healthcare. Another challenge is securing big data which requires a centralised storage, bringing into the picture the healthcare-specific clouds. Similar solutions already exist, and the cloud-based healthcare platforms are already operational in Google, Amazon and Microsoft, enhanced with high-performance computing and AI. The risks remain however on the edge of the network, with low-cost Internet-of-things (IoT) devices and systems, which are increasing on a large scale. Since the risk surface is increasing at the edge, to reduce the number of cyber attacks, we need to shift the cyber-risk analytics to the edge of the network.

The levels of digital healthcare services will continue to increase, from digital front door and telehealth, to augmented reality, virtual reality, and robotic surgery, creating urgency in identifying the correct level of cybersecurity requirements and the risk of these complex and coupled systems. In summary, COVID-19 has provided a powerful incentive for adopting and scaling-up of technological solutions at speed. But these solutions come with compromises and risks and potential unintended consequences from their implementation at this scale and speed.

## Methodology for integrating artificial intelligence and real-time data with edge analytics of health devices

The existing methodologies are focused on addressing individual aspects of the COVID-19 pandemic in isolation (e.g. tracking, managing, securing, vaccinating), while COVID-19 has shown us that we need a combination of approaches applied in a synchronised approach that supports and enhances the overall process. To secure the complex and coupled healthcare systems, we need to start constructing combinations of possible solutions—learning from the COVID-19 pandemic. One such solution is to refer to the concept of digital modernity [13] and smart manufacturing (i.e. Industry 4.0) [14] and develop a dynamic and self-adapting system for predictive edge analytics of health devices [15], supported with artificial intelligence and real-time data. Such AI system should be based on deep learning algorithms, mathematical principles and quantitative data. This approach would intersect knowledge from AI, healthcare, supply chains, economics, risk assessment, and edge computing. By applying an interdisciplinary multi-method, this system would also record a snapshot in time and collect a diverse set of data on COVID-19, which can be reused for Disease X by future researchers long after COVID-19 is gone, promoting the development of a standardised COVID-19 open data sources [16]. The multi-method interdisciplinary research does not mean that the proposed system should present incremental research, but quite the opposite. According to current data, COVID-19 presents once in a century opportunity to study a global pandemic of this magnitude. Hence, by designing a system based on applying a multi-method interdisciplinary research, the emerging system would present novel knowledge.

Since COVID-19 is a rare event, the system would need to be constructed with an iterative methodology, building upon knowledge developed through individual phases. For this approach to be successful, the methodology would need to be designed with the different phases organised in six steps (i.e. six cycles), namely ‘**prepare’, ‘measure’, ‘adapt’, ‘predict’, ‘defence’ and ‘improved’** the readiness for a Disease X event.

The process and activities in the workflow include using data from COVID-19 observations to **prepare** narratives for a Disease X event; using data from COVID-19 to **measure** and quantify the primary and secondary risks from a disease × event, using existing digital technologies (e.g. Industry 4.0, Internet of Things) to **adapt** the vaccine production and supply chains; using **predictive** algorithms for assessment of failures and losses; using AI algorithms for **securing (defending)** the healthcare system; and using existing AutoML methods for **improving** such AI algorithms.

To construct categorisations of concepts related to AI, real time data, and edge analytics, we first conducted a quantitative search for research data records on the Web of Science core collection. This resulted in a large data set, which we firstly analysed with the Web of Science results analysis tool. Secondly, we performed bibliometric analysis of the data records with R studio, applying the bibliometrix analysis package. This presented various visualisations of the data, which are extracted and categorised in a summary map (Table 1).

**Table 1:** Summary map of the methodology for integrating artificial intelligence and real-time data with edge analytics of health devices

Phase (P) of the methodology	Novel scientific approaches and methodologies required for managing Disease X
P_1_: How can we **prepare** for Disease X?	Important methodological challenges: create narratives of alternative mental health (i.e., digital) therapies used during COVID-19
	Novel concepts and methodological approaches: create digital records of COVID-19 alternative mental health (i.e. digital) therapies used during COVID-19
	Methodological output: develop a method for preserving the mental health during lockdowns as a coping mechanism and alternative to physical social life [17]
P_2_: How can we **measure** the risks from Disease X?	Important methodological challenges: develop AI that can operate on healthcare edge devices
	Novel concepts and methodological approaches: create new AI algorithms specific for cybersecurity of healthcare systems—based on a range of Disease X characteristics
	Methodological output: algorithms for predictive and dynamic risk quantification in the healthcare system with real-time intelligence
P_3_: How can we **adapt** the healthcare system for Disease X?	Important methodological challenges: construct adaptive algorithms for securing the vaccine supply chain during a Disease X event—e.g. integrate vaccine production and supply chains with the concept of Industry 4.0 and use of new technologies, such as 3D printing, drones
	Novel concepts and methodological approaches: develop adaptive digital supply chain solutions for the healthcare system (e.g. use of drones, autonomous vehicles, 3D printers)
	Methodological output: construct alternative vaccine delivery systems based on new technologies—for resolving shortages of supplies in critical times
P_4_: How can we **predict** the loss from Disease X?	Important methodological challenges: forecast the potential loss from Disease X in combination with other events—AI cyber attack, e.g. apply existing risk assessment models: NIST, FAIR
	Novel concepts and methodological approaches: build a mathematical model for predicting the primary and secondary loss (e.g. adapt the factor analysis of information risk model)
	Methodological output: construct scenarios and prevention strategies for AI cyber attacks on the healthcare system during Disease X crises
P_5_: How can we use AI for cyber **defence** during Disease X?	Important methodological challenges: Map the future cyber-attack surface in healthcare systems
	Novel concepts and methodological approaches: build a new AI algorithm that can prevent active and passive reconnaissance in healthcare devices operating on edge technologies
	Methodological output: develop algorithms that will enable the healthcare systems to continue operating even when compromised
P_6_: How can we teach AI to train new and **improved** AI algorithms for Disease X?	Important methodological challenges: Create AI algorithm can improve the existing algorithms (at speed) used in healthcare systems
	Novel concepts and methodological approaches: train algorithms how to decode the virus characteristics to predict the virus behaviour in fast changing events and to assist the healthcare system to anticipate a future Disease X event
	Methodological output: develop algorithms that will test and adapt to the specific requirements of healthcare systems, e.g. existing AutoML already provides multiple autonomous solutions

**Fig. 1:**
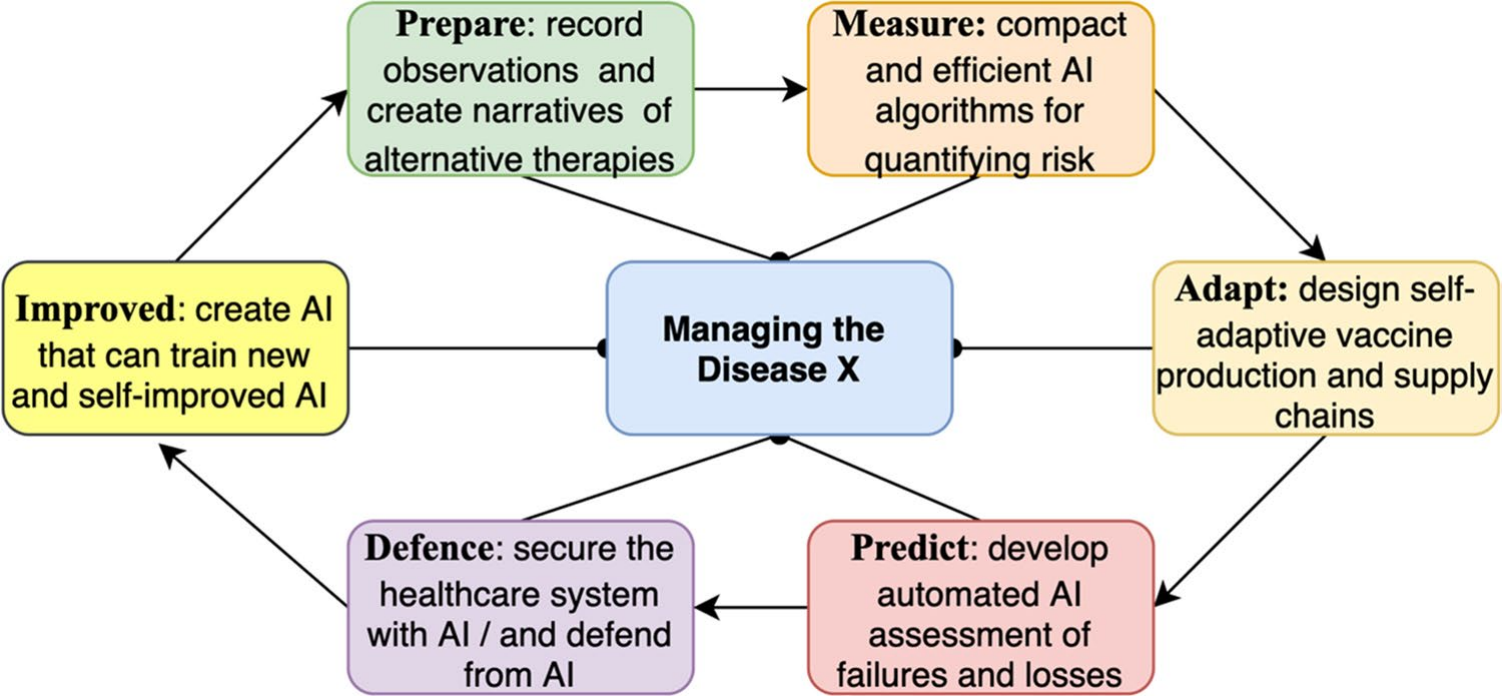
Workflow of the methodology for integrating AI in healthcare systems

The iterative methodology for integrating AI in healthcare systems described in Fig. 1 is best suited for studying unknown and unpredictable topics, because all of the phases (cycles) will benefit from the knowledge synthesised in individual cycles. For example, the ‘preparing’ phase would enhance our understanding of the risks and values that need to be measured, defended and improved. The other five phases would enable the ‘preparing’ cycle to record data analysis on COVID-19 that will not otherwise be available. This methodology is designed with a gradient of risk appropriate to the grade of gain, by combining conventional with novel research approaches in preparing with qualitative techniques, measuring and predicting with mathematical principles, adapting with engineering strategies, and defending and improving with computer science tools. To clarify the new approach, the complete and detailed methodological work plan is described in a summary map (Table 1).

The new methodology outlined in Table 1 is grounded on the postulate that support services in many sectors, including healthcare, will evolve dramatically by the time we are faced with Disease X—e.g. with the integration of AI in 5G networks. Therefore, the methodology proposes the development of new AI algorithms that would be able to predict and respond to user traffic, and provide 24-h security access, while preserving energy and lowering cost, e.g. by turning edge devices into sleep mode when demand is low. These advantages come at a risk, because AI is not invincible and can be manipulated. AI can be tricked into labelling dangerous software or user behaviour as safe, or the other way around. AI can be really helpful in detecting some weaknesses and potential threats immediately, such as weak passwords—which account for over 80% of data breaches.

The case is very different from biometric authentication, which cannot be changed. For example, if AI is authorised to use and control biometric data, and the data falls in the wrong hands, this can be used for surveillance and other infringements of privacy. AI has enabled companies to collect, process, and analyse much more data than we could do otherwise. This accumulation of data increases the attack surface and leads to further deterioration of privacy and security [18]. Data accumulation is predominately happening in large tech companies, because AI technology is still experimental, and the cost of implemention is too high for small companies. This just described one possible scenario for future large-scale cyber-attacks. The data accumulation (at specific data centres) has created significant incentive and benefits for hackers to break into the AI-secured operations, with a motive to manipulate the AI and infiltrate malware that resembles a trusted software. In the same way that companies are using AI, hackers are also using the same methods to analyse failed attempts and improve future attacks.

## Analysing the proposed methodology

Since the proposed methodology is targeted at addressing multiple problems that emerged as a result of COVID-19 (e.g. healthcare capacity, security, medical supply chain bottlenecks), it is challenging to analyse the output of the proposed approach. Hence, the conceptual design has been selected as the most appropriate approach and analysed through case study research. However, individual solutions have already started to emerge, such as the ‘Linearfold’ prediction algorithm that can calculate and predict the secondary structure of the RNA sequence of COVID-19 in 27 s instead of 55 min.^1^ Google's Alphabet has also shared its predictions of coronavirus protein structures.^2^ Similarly, IBM, Amazon, Google and Microsoft have also provided the computing power of their servers for analysing datasets in epidemiology, bioinformatics and molecular modelling.^3^

**Fig. 2:**
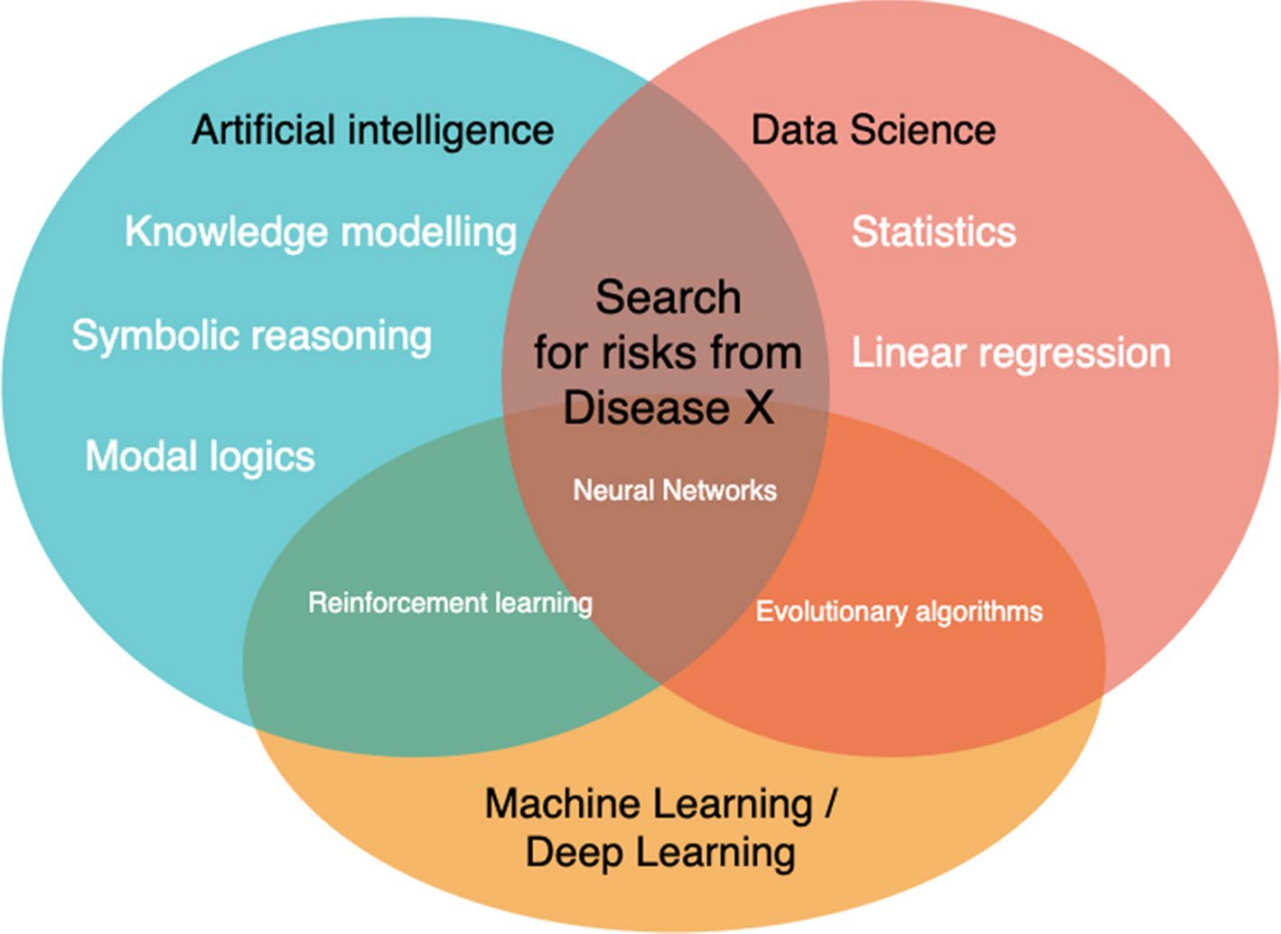
Foundations of the new methodology for integrating AI in healthcare systems

The summary map of the methodology (Table 1) extends into the development of novel approaches for measuring the risks emerging from COVID-19, e.g. working from home during lockdowns. The novelty of this approach is the integration of AI algorithms with established statistics methods (Fig. 2): (1) distribution; (2) probability; (3) Bayesian methods.

The current state of the art in healthcare systems’ cyber-risk impact assessment is based on manual calculations using the Bayesian approach. This means that when new data are added, a new manual statistical analysis of the entire dataset needs to be performed, and this process is completely necessary, because the results can be quite different depending on the new data added. Therefore, the previous forecast could be proven completely wrong, and this creates a lot of work (i.e. because currently is conducted manually). To reduce this complexity, the proposed methodology in Table 1 is grounded on automated approach (i.e. the Bayesian approach), applying discrete binary (i.e. Bernoulli) probability distribution to determine data breaches in a given time and the probability of a system going down; and to apply continuous probability distribution to determine the range and impact of a given event (size and duration). The aim of the new methodology is to guide the development of AI algorithms that can operate on healthcare edge devices to detect and resolve Disease X anomalies before they turn into faults. There are operational AI algorithms that can function on edge devices [19] and these algorithms need to be tested for detecting Disease X anomalies.

### Technological and algorithmic requirements for the proposed methodology

The six phases (cycles) of the methodology conceptualises the construction of a more compact and efficient version of AI algorithms, because the key to outperforming the hackers in a fast-changing Disease X event is to increase research on deep learning algorithms and to deploy AI in edge devices, IoT, drones [20]. This is not only required for cybersecurity, but also for vision, speech, and more general healthcare. The problem is that those devices have very low memory and current AI algorithms cannot run on those devices. To make this possible, we need faster and more efficient processing—but not necessarily hardware. The current state-of-the-art assumes that for better, faster and more efficient processing, we need a better hardware. There could be an algorithmic solution to this, for example, our brain operates at 20 W, while a single GPU operates at 300 W. This means that probably there are more efficient versions of the algorithms that we use. The second problem is that the current state of the art assumes that to deploy AI in edge devices, we need more memory in edge devices. There could be algorithmic solution to this as well. The current AI neural networks are based on dense representations, such as dense multidimensional metrics called ‘tensor’. Our brain is extremely sparse, compact and efficient. We will try to develop AI algorithms that are more compact and efficient, so that we can deploy them on edge devices. The methodology emerging from this article (Table 1) proposes the development of a series of new algorithms and building upon each algorithm to reach a state where AI algorithms are sparse, compact and efficient. As outlined in the summary map, the emerging methodology proposes undertaking experimental developments in research on sparse, compact and efficient AI algorithms for a very specific function—to be applied on edge devices to measure the emerging risk of a disease × event (Table 2).

**Table 2:** Structure of the methodology for integrating AI in healthcare systems

	Outline of the problems (P), proposed solutions (S), and expected results (R) from integrating AI in healthcare systems					
	P_1_: p**repare** for Disease X	P_2_: m**easure** risks from Disease X	P_3_: a**dapt** for Disease X	P_4_: p**redict** the loss from Disease X	P_5_: d**efence** during Disease X	P_6_: i**mprove** AI for Disease X
P	Prolonged lockdowns	Cyber-risk quantification	Securing the vaccine supply chain	Primary and secondary loss	Increased cyber-attack surface	Training new AI algorithms
S	Digital narratives	New design of AI neural networks	Adaptive digital supply solutions	Scenarios and prevention strategies	AI algorithms for cyber defence	Train algorithms to decode cognition
R	Method for preserving mental health	AI algorithms based on compact representations	Alternative vaccine delivery systems	Mathematical model	Systems resistant to compromises	Algorithm writing AI algorithms

## Discussion

The current trend in AI-driven chatbots for healthcare has produced numerous bots, e.g. Florence; Infermedica; Buoy Health. Such AI-driven bot could take advantage of big data to launch an automated social engineering attack. The attacker’s bot could interact with the victim in a way that sounds legitimate, using direct references to results obtained from OSINT queries. The methodology presented in this article can identify how OSINT queries could allow the bot to ‘learn’ new patterns and become more efficient. To achieve that, the methodology would need to synthesise data with modern reconnaissance tools, to construct a map of the attack surface in healthcare systems, e.g. (cold chain) supply chain. The adversarial AI system could potentially modify the attack and victim interaction with things supporting its narrative. Similar to current botnets used for denial of service (DoS) attacks, threat actors could have millions of AI-based bots looting the Internet to perform attacks. Such bots, operating at times of global emergencies, could create a significant loss of life.

## Conclusion

This article presents a novel methodology for integrating functional AI in healthcare systems, grounded on designing more compact and efficient algorithms that can perform analytics on healthcare edge devices. Through designing the concepts for the creation and enablement of autonomous devices that can operate on the edge of the networks. This article described a design process for using artificial intelligence and edge devices as an early warning system for virus abnormalities. The article presents a new methodology for integrating artificial intelligence in digital healthcare systems for managing future waves of COVID-19 and a future Disease X event. The proposed methodology is conceptual and with a focus on solutions based on low memory Internet of Things devices operating at the edge of the network. The conceptual methodology can be used to prepare and adapt to a variety of potential Disease X events, and to measure cyber risks and predict the loss from Disease X, to prepare defence (e.g. from cyber attacks, from medical equipment production and supply chain bottlenecks) and to learn from COVID-19 failures (e.g. failures in detection and diagnosis) to improve artificial intelligence algorithms for future Disease X events.

### Limitations and further research

Since medical and health systems are based on ‘care’, this article distinguishes between human healthcare and AI systems that do not understand care. Current AI systems do not possess consciousness, these systems cannot care and understand, or be intelligent and creative in the same way as humans are. Therefore, in this article, we reviewed the functional intelligence and not consciousness of the AI systems. All of the data on COVID-19, required for this research, were acquired from publicly available open access data sources.

## Data Availability

All data and materials included in the article.

## Footnotes

1 https://www.technologyreview.com/2020/03/11/905366/how-baidu-is-bringing-ai-to-the-fight-against-coronavirus/.

2 https://deepmind.com/research/open-source/computational-predictions-of-protein-structures-associated-with-COVID-19.

3 https://techcrunch-com.cdn.ampproject.org/c/s/techcrunch.com/2020/03/22/ibm-amazon-google-and-microsoft-partner-with-white-house-to-provide-compute-resources-for-covid-19-research/amp/.

## Code availability

N/A—no code was developed; code was however used for running the R Studio analysis.
